# A Case of Asymptomatic Cronkhite-Canada Syndrome Diagnosed by Endoscopic and Pathological Findings

**DOI:** 10.7759/cureus.88876

**Published:** 2025-07-28

**Authors:** Yusaku Yokotani, Hiroaki Kitae, Mika Mazaki

**Affiliations:** 1 Department of Gastroenterology, Omihachiman Community Medical Center, Omihachiman, JPN; 2 Department of Gastroenterology, Asahi University Hospital, Gifu, JPN

**Keywords:** cronkhite-canada syndrome, gi polyposis syndrome, hypoalbuminemia, prednisolone, rare case report

## Abstract

Cronkhite-Canada syndrome (CCS) is a rare, non-hereditary disease characterized by specific symptoms, ectodermal abnormalities, endoscopic findings, and pathological examination. We report a unique case of a 39-year-old man with suspicious CCS. Although the blood test showed iron-deficiency anemia and hypoalbuminemia, he exhibited no symptoms, including those related to CCS itself and anemia and hypoalbuminemia. According to the result of endoscopic and pathological examination, he was diagnosed with possible CCS. While following the patient’s medical course, the serum albumin level gradually declined. Six months after the diagnosis, oral corticosteroid therapy was initiated to prevent disease progression. Consequently, the patient achieved a favorable clinical course without developing any symptoms. This case highlights the importance of considering CCS even in the absence of typical symptoms and suggests that therapeutic intervention may be beneficial even in asymptomatic presentations.

## Introduction

The number of reported cases of Cronkhite-Canada syndrome (CCS) was estimated to be approximately 500 in 2019, with around 75% originating from Japan [1,2]. It is a non-hereditary disease characterized by diffuse gastrointestinal polyposis and ectodermal abnormalities. The diagnosis of CCS is based on the presence of diffuse gastrointestinal polyposis predominantly involving the stomach, small intestine, and colon, accompanied by characteristic ectodermal changes such as alopecia, skin hyperpigmentation, and nail dystrophy. Additional clinical features commonly include chronic diarrhea, weight loss, and hypoalbuminemia. The diagnosis requires the exclusion of other polyposis syndromes with similar endoscopic or histologic findings [3].

Referring to the Japanese nationwide survey, chronic diarrhea is reported in approximately 78% of patients with CCS. Moreover, ectodermal abnormalities, such as alopecia, onychodystrophy, and skin hyperpigmentation, are observed in over 70% of cases, with 91% of those patients presenting at least one of these characteristic findings [4].

Although the etiology of this disease remains unclear, autoimmune, infectious, drug-related, or psychological factors have been implicated in its pathogenesis [5,6]. Among these, the possible involvement of immune-mediated mechanisms may explain why oral corticosteroid therapy has shown clinical efficacy and appears to improve the natural course of CCS, as demonstrated in previous studies [4,7]. However, there are not a few recurrence cases, and additional treatment is sometimes needed, such as cyclosporine [8]. Additionally, the concurrence of malignant tumors of the gastrointestinal tract is more likely than in the general population. Therefore, early induction of remission, maintenance treatment, and regular follow-up are needed [4]. The incidence of cancer after polyps has disappeared is unknown, but regular endoscopic surveillance is recommended [4,9].

Diagnosing CCS before the onset of severe symptoms may prevent malnutrition and avoid irreversible complications. However, because of its rarity and nonspecific presentation, CCS is often difficult to diagnose promptly. Endoscopic and histopathological findings are key to diagnosis, but in most reported cases, patients present with overt clinical symptoms. Here, we report a unique case of CCS diagnosed in an asymptomatic patient based on endoscopic and histological findings.

## Case presentation

**Figure 1:**
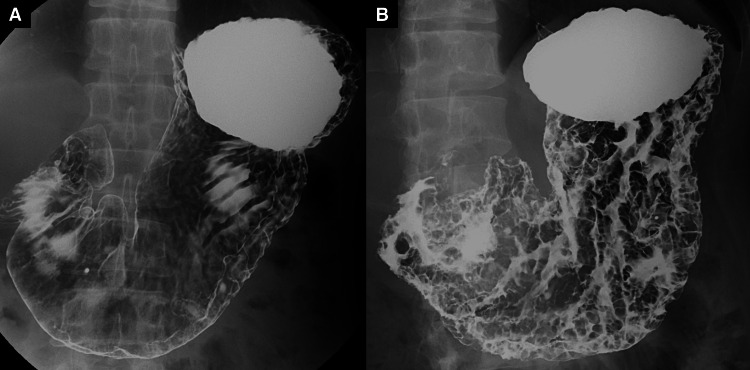
Fluoroscopic images of upper gastrointestinal series (UGI) series A) UGI series, which was examined one year before the diagnosis, revealed no major findings. B) The latest UGI series revealed multiple radiolucent lesions and a thickened, irregular gastric wall.

The patient was a 39-year-old Japanese man with no significant past medical or family history. He underwent a medical checkup one year ago, but the results of the blood test and upper gastrointestinal (UGI) series were normal (Figure 1A). When he received the annual checkup one month before the reference to our hospital, the result of the blood test showed anemia and hypoalbuminemia. Additionally, the latest UGI series revealed the diffuse irregularity of the gastric wall (Figure 1B). Consequently, he was referred to our hospital for further examination.

**Figure 2:**
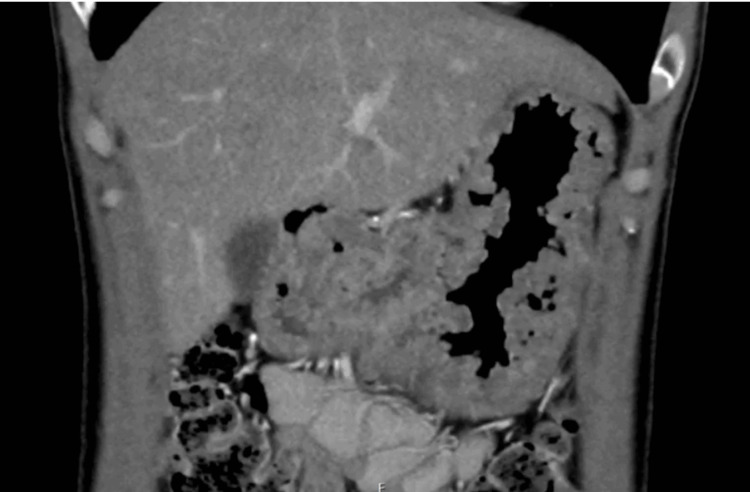
The image of computed tomography (CT) finding CT reveals wall thickening of the stomach and numerous polyp-like protrusions.

**Figure 3:**
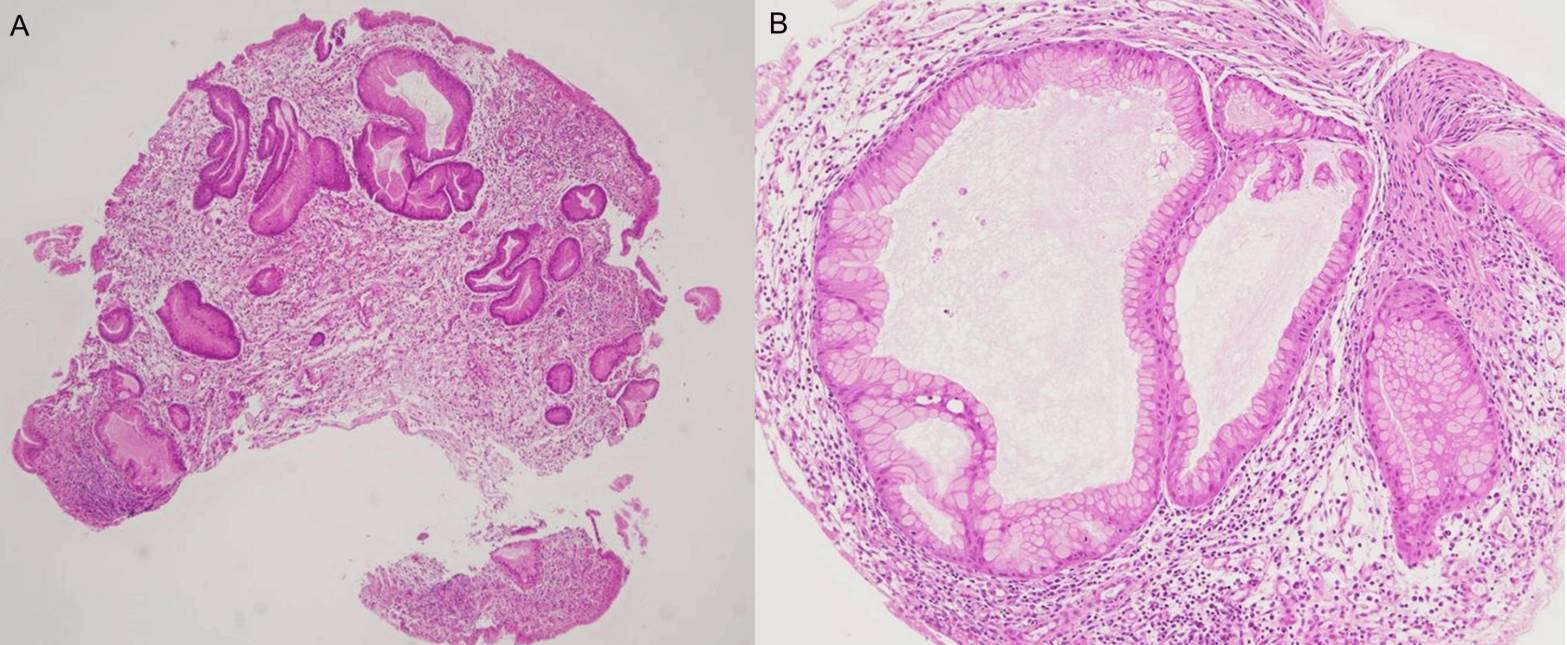
The images of pathological findings Histological examination of biopsy specimens obtained from the gastric polyps. Hematoxylin and eosin staining: (A) ×1, (B) ×10.

Medical interview and physical examination revealed no specific CCS-related symptoms or findings, including weight loss, diarrhea, abdominal pain, dysgeusia, and ectodermal changes. Blood tests showed hypoalbuminemia and iron-deficiency anemia (Table 1). Computed tomography (CT) revealed wall thickening of the stomach and numerous polyp-like protrusions in the stomach (Figure 2). More than 50 sessile-type (Is) polyps, measuring up to 20 mm in diameter, were observed predominantly from the gastric antrum to the lower gastric body. Polyps exhibited edema and erythema, and the intervening mucosa showed edematous changes. The biopsy of the polyp showed cystically dilated glands and foveolar hyperplasia associated with inflammation and edema of the lamina propria (Figure 3). Additionally, non-neoplastic changes were observed in the polyps. Although there was no obvious eosinophil infiltration, these findings can distinguish CCS from other gastrointestinal polyposis syndromes. No major abnormal findings were observed in the duodenum and esophagus. Additionally, there were no notable abnormalities on total colonoscopy (TCS). CCS is a disease in which polyps are diffusely present in the digestive tract, which distinguishes it from other inflammatory diseases, including Crohn’s disease/ulcerative colitis (UC)/tuberculosis (TB)/Menetrier’s disease. Endoscopic and pathological findings are the key to clarifying the difference between CCS and other inflammatory diseases of the GI tract.

**Table 1:** Blood test CRP: C-reactive protein; MCV: mean corpuscular volume; TIBC: total iron-binding capacity; UIBC: unsaturated iron-binding capacity

Laboratory Parameter	Result	Reference Range	Unit
Albumin	3.9	4.1-5.1	g/dL
Hemoglobin	9.6	13.7-16.8	g/dL
MCV	65.8	83.6-98.2	fL
Serum iron	8	40-188	mEq/L
UIBC	403	170-250	μg/dL
TIBC	411	180-270	μg/dL
Ferritin	6.9	42-326	ng/mL
CRP	0.05	<0.3	mg/dL

The patient was diagnosed with possible CCS based on blood tests, endoscopic findings, and pathological results. The severity of the disease was moderate based on CCS severity scoring (Table 2). The initiation of corticosteroid treatment was also considered. However, because he had no symptoms, we decided to observe the medical course carefully without steroid treatment. Following the diagnosis, oral iron supplementation was administered for two months. An increase in hemoglobin levels was observed; however, the treatment was discontinued upon the patient’s request.

**Table 2:** Severity assessment criteria for Cronkhite-Canada syndrome Activity is evaluated by the total score for each item. Term definitions are as follows (cited from Atlas of Cronkhite-Canada Syndrome [10]): Disease activity: severe disease - item 2 ≥2 points and the total score ≥10 points; moderate disease - intermediate between severe and mild disease; mild disease - all items from 2 to 7 = 0 points and a total score ≤3 points. Therapeutic effects: improvement - total score decreased by 2 points or more; exacerbation - total score increased by 2 points or more. Clinical remission/relapse: clinical remission - total score ≤1; relapse - total score ≥2.

Item	Score	Criteria
1. Stool frequency/day	0	Normal for patient
	1	1-2 times/day more than normal
	2	3-4 times/day more than normal
	3	More than five times/day, more than normal
2. Serum albumin level	0	>3.5 g/dL
	1	≥3.0 to ≤3.5 g/dL
	2	≥2.5 to <3.0 g/dL
	3	<2.5 g/dL
3. Edema	0	None
	1	Mild (obvious pitting edema)
	2	Moderate (indistinct veins and bones)
	3	Severe (obviously swollen)
4. Doctor's overall evaluation	0	Normal (complete remission)
	1	Mild illness
	2	Moderate illness
	3	Severe illness
5. Weight loss (≥5 kg in six months)	0	None
	1	Applicable
6. Anemia (Hb <12 g/dL for men, <10 g/dL for women)	0	None
	1	Applicable
7. Blood in the stool	0	(−): None
	1	(+): Slightly mixed blood in less than half of the defecation
	2	(++): Obvious bloody stool in every defecation
8. Other clinical findings	-	Cutaneous manifestations (alopecia, onychodystrophy, or hyperpigmentation, plus 1 point if any of them applies)
		Dysgeusia
		Intussusception

**Figure 4:**
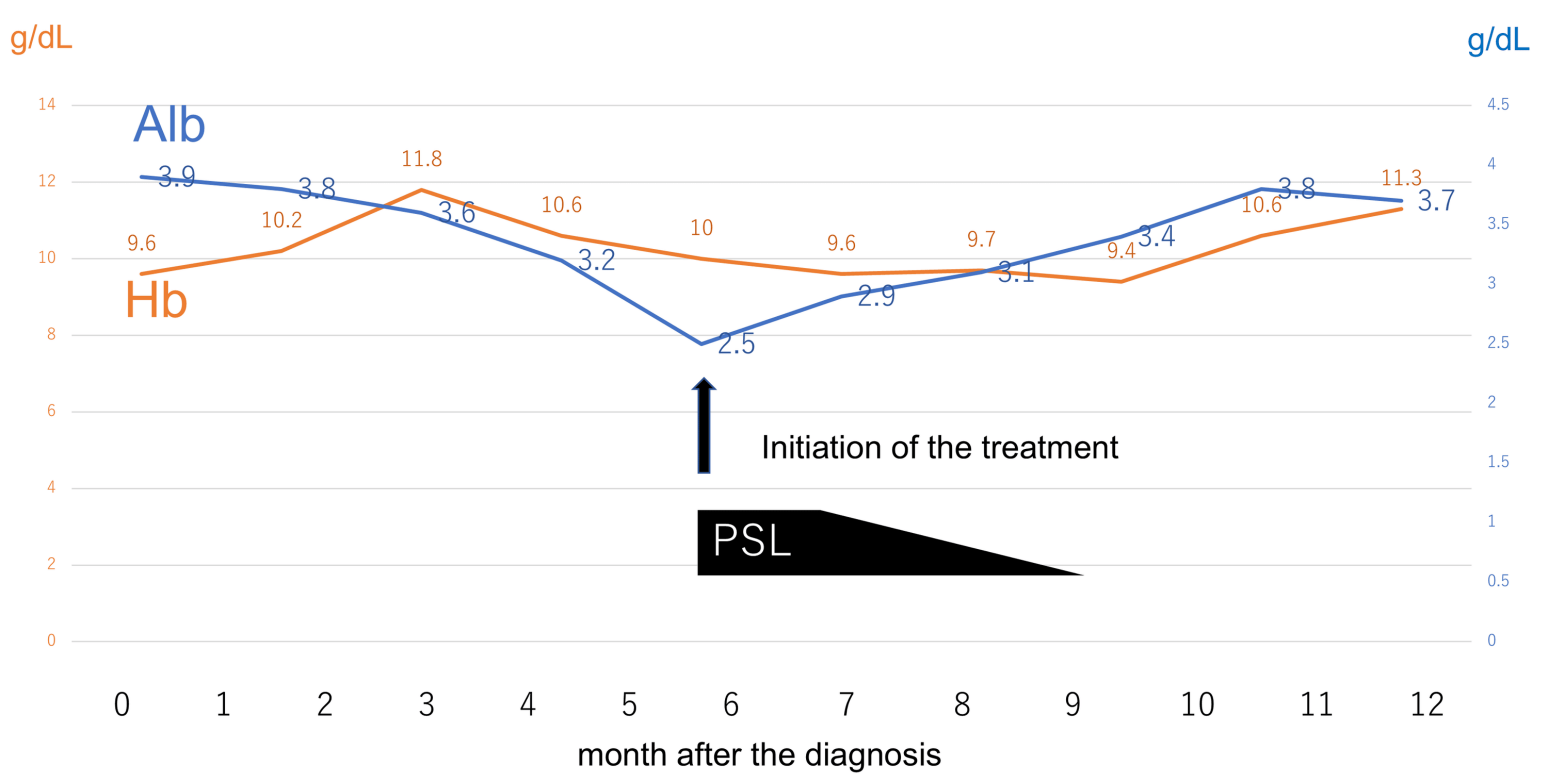
Time series of changes in albumin levels and Hb

The decision to initiate corticosteroid therapy was based on the progressive decline in serum albumin (Figure 4), which is strongly associated with the severity of disease activity. Oral prednisolone (40 mg/day) was initiated six months after the diagnosis, at which point the serum albumin level had decreased to 2.5 mg/dL and the severity scoring increased by two points, indicating an exacerbation of disease activity. Although there was no TB preventive therapy, Bactrim prophylaxis had been used during the duration of steroid usage. One month after initiating treatment, blood tests showed an increase in albumin levels (2.9 g/dL). Additionally, follow-up gastrointestinal endoscopy confirmed the improvement of edema, friability, and hypertrophic areae gastric. These findings are considered important factors based on endoscopic disease activity (Table 3).

**Table 3:** Endoscopic disease activity standards Cited from Atlas of Cronkhite-Canada Syndrome [10].

Severity	Findings
Mild	Erythema
Moderate	Edema, friability (contact bleeding), hypertrophic areae gastricae
Severe	Hypertrophic changes, spontaneous bleeding, white mucous exudate

**Figure 5:**
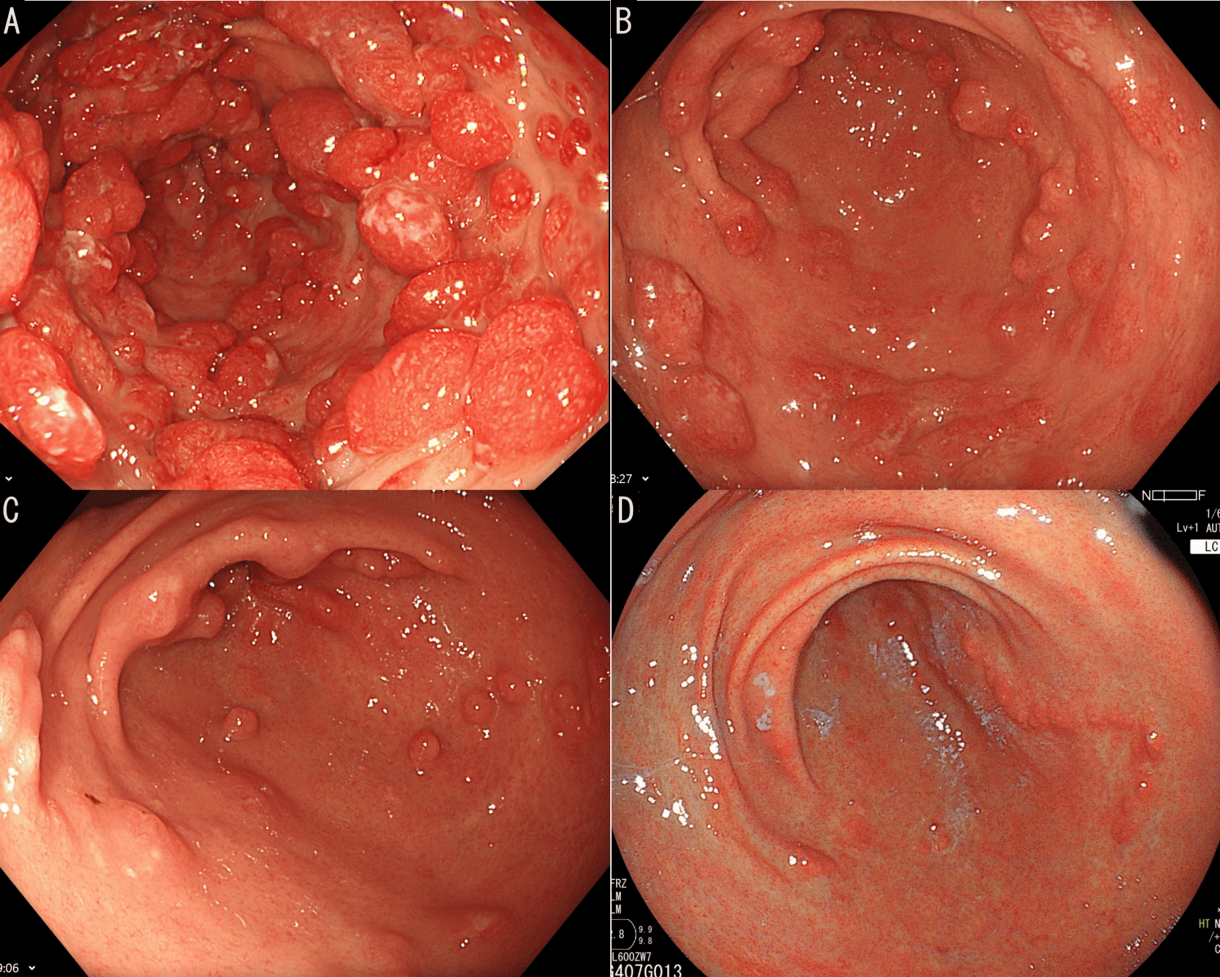
Time series of changes in mucosa and polyps at the antrum A) Two months before the initiation of the treatment. B) Five months after the initiation of the treatment. C) Seventeen months after the initiation of the treatment. D) Twenty-nine months after the initiation of the treatment.

Serial follow-up of endoscopic findings revealed the shrinkage of the numerous polyps and the gradual transition of the intervening mucosa to normal mucosa (Figure 5). Because a too-rapid steroid dose reduction can be associated with early relapse, the steroid dose was tapered gradually while checking the endoscopic findings and serum albumin levels. When two months and one week had passed, steroid treatment was discontinued. The patient has been regularly followed up with blood tests and GS, and the latest follow-up inspection was two years and three months after the termination of treatment. There was no aggravation tendency, almost all polyps disappeared, and the intervening mucosa was normal. Additionally, no gastric tumor had been detected.

## Discussion

In our case, numerous reddish and edematous polyps were observed by GS, mainly in the distal part of the stomach, which looked like juvenile hamartomatous polyps or hyperplastic polyps, and the intervening mucosa showed edematous changes. The main cause of hypoalbuminemia is thought to be protein leakage from the gastrointestinal mucosa. As for iron deficiency anemia, in this case, it can be thought that chronic bleeding from polyps or intervening mucosa was the cause. Histological examination of the polyp revealed cystic dilatation of glands accompanied by inflammation and edema within the lamina propria. These findings are characteristic of CCS. The present patient had no specific symptoms, and before the typical symptoms had appeared, he was diagnosed with possible CCS from these endoscopic and pathological features, and hypoalbuminemia. The timing of initiating treatment was determined depending on the serum albumin level, because hypoalbuminemia is considered an important factor in the classification of disease severity [10].

In terms of the treatment, oral corticosteroids (30-49 mg/day) are considered the most effective treatment for active disease [4]. Given that rapid tapering of corticosteroids can be associated with early relapse, the steroid dose must be gradually reduced with close monitoring of endoscopic findings and CCS severity scoring [4]. In this case, the administration was started at 40 mg and gradually tapered so that it could be completed within two months. This is because the condition is clinically divided into good early clinical remission or good early clinical response based on responsiveness to two months of steroid usage [10]. This case is classified into the good early clinical response group. Some cases reported recurrence of CCS, and additional treatment, such as cyclosporine usage, was needed [8]. Consequently, patients should be monitored carefully after the termination of the treatment.

The more crowded the polyps are and the brighter the color of the polyps is, the more significant the hyperemia and edema are in the mucous membrane around the polyps. Additionally, the clinical symptoms tend to appear strongly [11]. Approximately 65% of gastric polyps are considered hamartomatous polyps, and the other polyps are hyperplastic. The majority of gastric polyps are smaller than 20 mm, and their morphology is mainly sessile. Colorectal polyps are mainly hamartomatous and adenomatous [11]. Additionally, CCS polyps can be distributed on the mucosal surface like carpets or clusters. Interstitial edema and inflammatory edema were also found in the mucosa between the polyps. These characteristics are often not found in other forms of gastrointestinal polyposis and may, therefore, help distinguish the different types [11,12]. CCS gastric polyps and intestinal polyps have a high malignant transformation rate of more than 15% [4,11]. The incidence rate of cancer following the disappearance of polyps is unknown. However, considering its high mortality, malignant-transformation possibility, and the cases of recurrence after commencement of the treatment, early diagnosis, induction of therapy, and regular follow-up are necessary [4,7-9]. In our case, there were no characteristic polyps of CCS or inflammatory changes in the colon. Therefore, CS is not routinely indicated. However, revaluation of GS and CS may be warranted in the event of disease recurrence. The patient has undergone regular gastroscopic evaluations every six months and blood tests every three months to ensure no relapse. According to the previous report, regular surveillance endoscopy, preferably at intervals of one year or less, is recommended for monitoring mucosal disease activity and for the detection and removal of adenomas or other premalignant lesions [13]. Examination of annual GS is considered in this case.

## Conclusions

CCS is a sporadic disorder that is infrequently encountered in clinical practice. To achieve a favorable treatment outcome, it is essential to recognize the typical endoscopic and clinical features of CCS. Also, this disease should be considered as one of the differential diagnoses during health checkups and in daily clinical practice, not only by gastroenterologists but also by a broad range of clinicians.

Additionally, its prognosis and clinical course may be influenced by the timeliness of diagnosis and initiation of treatment. In our case, early therapeutic intervention was performed prior to the onset of characteristic symptoms, which appeared to induce remission, as evidenced by improvements in serum albumin levels and endoscopic findings. Even in asymptomatic patients, appropriate treatment may be possible by evaluating CCS severity scoring and endoscopic findings over time. In our asymptomatic case, albumin was a useful indicator of the disease’s progression and treatment efficacy. Reports of asymptomatic CCS patients are valuable, and it is hoped that similar cases will be reported in the future.
